# Does expanded artificial intelligence improve the prognostic value of myocardial perfusion imaging? A report from the NHLBI-sponsored women's ischemia syndrome evaluation (WISE)

**DOI:** 10.1186/1532-429X-15-S1-P273

**Published:** 2013-01-30

**Authors:** Mark Doyle, Gerald M Pohost, Leslee J Shaw, Diane A Vido, Sheryl F Kelsey, BD Johnson, William J Rogers, Geetha Rayarao, Barry L Sharaf, Carl J Pepine, Noel B Merz, Robert W Biederman

**Affiliations:** 1 Cardiac MRI, Allegheny General Hospital, Pittsburgh, PA, USA; 2 University of Southern California, Los Angeles, CA, USA; 3 Atlanta Cardiovascular Research Inst., Atlanta, GA, USA; 4 University of Alabama, Birmingham, AL, USA; 5 Brown University, Providence, RI, USA; 6 University of Pittsburgh, Pittsburgh, PA, USA; 7 University of Florida, Gainesville, FL, USA; 8 Cedars-Sinai Medical Center, Los Angeles, CA, USA

## Background

We previously introduced a model-based approach (Decisions Informed by Computed Entities [DICE]), a mathematical form of Artificial Intelligence (AI) to improve prognostic values of quantitative myocardial perfusion imaging (MPI). When DICE was applied to cardiovascular MRI (CMR) it improved prediction of MACE in women with suspected ischemic heart disease (IHD) compared to the conventional MPI interpretation. Our aim is to determine if expanded DICE modeling improves prognostic capability to predict MACE.

## Methods

Women (n=228), mean age 59±11 yrs, with suspected myocardial ischemia underwent MPI and cardiac function evaluation separately by CMR and SPECT. Primary endpoint: MACE defined as cardiovascular death, MI, and hospitalization for CHF. The MPI test and 3 comorbidities were assessed: diabetes (DM), hypertension (HTN), and inadequate stress (IS) during MPI testing. The prognostic value of each variable on overall survival was studied separately and in combination using Kaplan-Meier curves with log-rank tests. Boolean logic was used to determine final disease severity category (0-2).

## Results

Results During FU of 40+16mo, 22 women (10%) had MACE. The log-rank statistic for MACE prediction for MPI was 9 (p<0.005), for HTN was 12(p<0.001), for DM was 5 (p<0.05), and for IS was 2 (p=0.2). Combining MPI and HTN demonstrated the strongest prognostic value for predicting MACE (log-rank statistic 26, p<0.001) with 95% survival in pts without detectable disease vs. 70% survival in pts with positive MPI and HTN. Combining DM and IS provided further prognostic value in the MPI and HTN positive group with survival rates of 79% and 40% (p<0.05). For MPI, HTN, DM and IS, positive test results were assigned a 1 and negative results were assigned 0. Using Boolean logic, when categorizing disease in pts as the sum of (MPI AND HTN) + (DM AND IS) pts had score of 0 (mild), 1 (moderate), or 2 (severe), the Kaplan-Meier log- rank statistic over the three disease severity categories was 42 (p<0.001) with actual survival rates of 97%, 80% and 40% for increasing disease severity.

## Conclusions

Expanded DICE mathematical modeling via AI incorporated with MPI interpretation markedly increases prognostic values of MACE prediction in women with suspected IHD over standard MPI.

## Funding

NIH

